# Theranostics for Triple-Negative Breast Cancer

**DOI:** 10.3390/diagnostics13020272

**Published:** 2023-01-11

**Authors:** Hyeryeon Choi, Kwangsoon Kim

**Affiliations:** 1 Department of Surgery, Eulji Medical Center, Eulji University School of Medicine, Seoul 01830, Republic of Korea; 2 Department of Surgery, College of Medicine, The Catholic University of Korea, Seoul 06591, Republic of Korea

**Keywords:** theranostics, nanomedicine, immunotherapy, breast cancer

## Abstract

Triple-negative breast cancer (TNBC) is an aggressive subtype of breast cancer with poor prognosis. Current endocrine therapy or anti HER-2 therapy is not available for these patients. Chemotherapeutic treatment response varies among patients due to the disease heterogeneity. To overcome these challenges, theranostics for treating TNBC have been widely investigated. Anticancer material conjugated nanoparticles with target-binding ligand and tracer agents enable simultaneous drug delivery and visualization of the lesion with minimal off-target toxicity. In this review, we summarize recently FDA-approved targeted therapies for TNBC, such as poly-ADP-ribose polymerase (PARP) inhibitors, check point inhibitors, and antibody-drug conjugates. Particularly, novel theranostic approaches including lipid-based, polymer-based, and carbon-based nanocarriers are discussed, which can provide basic overview of nano-therapeutic modalities in TNBC diagnosis and treatment.

## 1. Introduction

Cancer is the second leading cause of death worldwide. For women, breast cancer accounts for almost one-third of all cancer diagnosis [1]. The incidence of female breast cancer in the U.S. has been slightly increasing due to continuous declines in the fertility rates [2]. Traditionally, breast cancer is classified as four molecular subtypes, such as luminal A, luminal B, HER-2 enriched, and triple-negative breast cancer (TNBC). TNBC, which account for 15–20% of all breast cancer, Ref. [3] is defined as lack of the expression of hormones receptors (estrogen receptor (ER) and progesterone receptor (PR)) and lack of amplification of human epidermal growth factor receptor 2 (HER-2). TNBC is known to be an aggressive subtype, which has a higher rate of BRCA1 mutation, recurrence, metastasis, and mortality than other subtypes [4,5]. Precise diagnosis is an essential prerequisite for improving prognosis. However, TNBC diagnostics are still relies on conventional mammography, ultrasonography, magnetic resonance imaging (MRI), and immunohistochemistry (IHC), which are modalities that have limitations in the non-specific contrast agents and possibilities of false-positive findings [6].

Nowadays, “precision” medicine is quite routinely applied in cancer therapy. Precision medicine is defined as the tailoring of treatment using molecular and genomic determinants to classify individuals into specific groups that differ in their susceptibility to a particular disease or their response to a specific treatment [7,8]. Molecular subtyping using multigene array and targeted therapies for breast cancer is a good example of precision medicine. However, TNBC patients cannot benefit from currently available endocrine therapy or anti HER-2 therapy due to their lack of receptors [9]. Chemotherapy with taxanes and anthracyclines is the only systematic treatment option for TNBC. However, the treatment response varies between patients because of the heterogeneity of the disease. High toxicity of chemotherapeutic agents and multidrug resistance are also obstacles to the management of TNBC [6,10]. Recent development of molecular and gene expression analysis has been revealed in the intertumor heterogeneity of TNBC. Lehmann et al. discovered six TNBC subtypes using gene expression profiling: basal-like 1 (BL1), basal-like 2 (BL2), mesenchymal (M), mesenchymal stem-like (MSL), immunomodulatory (IM), and luminal androgen receptor (LAR) [11]. These authors also reported different responses to chemotherapeutic and targeted agents according to cell line models representing each of the TNBC subtypes [12]. However, these molecular classifications have not been shown to improve survival in clinical practice. Identification of effective diagnostic and therapeutic targets are challenging task for TNBC treatment. 

Over the past decade, tumors have been recognized as a collection of proliferating cancer cells. The six conventional hallmarks of cancer focus on the characteristics of the tumor cells itself. They include sustaining proliferative signaling, evading growth suppressors, resisting apoptosis, enabling replicative immortality, inducing angiogenesis, and activating invasion and metastasis. However, recent studies have revealed that normal cells composing stroma contribute to tumorigenesis. The communication between cancer cells and stromal cells as well as immune cells induces TME alteration, which predisposes cancer cells to metastasis [13,14]. TME is made up of extracellular matrix (ECM), immune suppressive cells, and re-programmed fibroblasts, which help initiation and progression of TNBC. Therefore, TME is suggested to be a therapeutic target of TNBC [15]. 

“Personalized” medicine means specifically designed treatment for individual patients. The term “theranostics” is defined as the materials that combine both diagnostic and therapeutic capabilities, which was first coined by Funkhouser in 2002 [16]. The concept of simultaneous delivery of drugs and contrast agent to the target site is expected to contribute to the personalization of current medicine. By employing nano-carriers, which were made from various materials such as polymers, lipids, nucleic acid, proteins, carbon, and metals including micelles and liposomes [17,18], theranostics are becoming closer to real-world practice. Anticancer drug conjugated nanoparticles with target-binding ligand and tracer agents enable accurate and efficient drug delivery as well as visualization of the lesion, with minimal toxicity to healthy tissues. Single nanoparticles can be conjugated with various functional materials at same time, such as targeting molecules, therapeutic agents, fluorophores, and/or radioisotopes. The therapeutic agents are not only chemo drugs but also therapeutic genes (e.g., siRNA), photothermal agents, radiosensitizers, and immunostimulants [19]. 

There are no FDA-approved theranostics for TNBC yet, but the growing need for theranostics is evident in TNBC treatment. In our review, we focus on the current precision medicine and theranostic tools that are developing. 

## 2. Precision Medicine for Triple-Negative Breast Cancer

### 2.1. Poly-ADP-Ribose Polymerase (PARP) Inhibitors (PARPi)

Poly-ADP-ribose polymerase (PARP) is an enzyme involved in single-strand break (SSB) DNA repair and genomic stability. The protein encoded by BRCA1/BRCA2 also plays an essential role in DNA repairing by the homologous recombination pathway. As a result, tumor cells with BRCA1/2 mutation present homologous recombination deficiency, which leads to vulnerability to SSB [20]. For these cells, inhibition of PARP results in accumulation of SSBs, which eventually leads to cell death. This mechanism is called synthetic lethality [21]. Gonzalez-Angulo et al. reported a 19.5% incidence of BRCA1/BRCA2 mutations in TNBC patients [22]. Some of the BRCA1/2 wild-type TNBC also shows the biological characteristics of deficiency in the homologous recombination pathway [23]. Therefore, PARPi are widely investigated for the TNBC treatment. 

In preclinical study, Hastak et al. reported greater sensitivity of TNBC to PARPi compared to non-TNBC [24]. PARP inhibitors’ improvement of progression-free survival (PFS) and overall survival (OS) has been verified by several clinical trials. The OlympiAD trial reported significant benefit of PFS (7.0 vs. 4.2 months, *p* < 0.001) of Olaparib treatment over standard chemotherapy in HER2-negative metastatic breast cancer patients with a BRCA 1/2 mutations. More than 40% of enrolled patients in the Olaparib group were TNBC. However, this trial was not powered to detect a difference in OS (19.3 vs. 17.1 months, *p* = 0.513), which might be confounded by subsequent treatment crossover after discontinuation of Olaparib [25]. For HER2-negative early breast cancer with BRCA1/2 germline mutations, a recent phase III OlympiA trial reported significant longer DFS (87.5% vs. 80.4%, *p* < 0.001) in the adjuvant Olaparib group compared to the placebo group [26]. The FDA approved Olaparib for the treatment of patients with BRCA-positive, HER2-negative metastatic breast cancer in 2018. This is the first approved targeted agent for TNBC. 

Each of the PARPi has various potency and cytotoxic effects. Talazoparib is one of the most potent and cytotoxic PARPi, which has a strong catalytic PARP enzyme inhibition and PARP-trapping potential. EMBARCA was the phase III randomized clinical trial that compared the PFS of talazoparib versus physician’s choice treatment in advanced breast cancer with germline BRCA1/2 mutations. Median PFS was 8.6 months in the talazoparib arm compared to 5.6 months in the standard therapy arm (*p* < 0.001) [27]. Based on this study, talazoparib was also approved for the treatment of patients with HER2-negative locally advanced or metastatic breast cancer with BRCA mutations in 2018. 

Several studies have investigated the benefit from PARP inhibitors in combination with chemotherapy in TNBC. Veliparib acts through inhibition of PARP without trapping of PARP onto DNA [28]. Since PARP trapping has been known to related with myelosuppression, which results in cytopenia such as anemia, thrombocytopenia, and neutropenia [29], veliparib is considered as a more suitable PARP inhibitor than other PARPi in combination with platinum-based chemotherapy. In a BROCADE 3 phase III randomized, placebo-controlled clinical trial, veliparib in combination with carboplatin and paclitaxel showed significant improvement in PFS (14.5 months vs. 12.6 months, *p* = 0.0016) in patients with BRCA mutation-associated HER2-negative advanced breast cancer [30]. 

Further studies are ongoing for enhancing synthetic lethality and reducing resistance of PARPi. The combined use of DNA methyltransferases inhibitors or β-lapachone represented a synergistic anti-tumor effect in TNBC xenograft models [9,31,32]. The mechanisms of PARPi resistance have been presented for few categories: restoration of homologous recombination, replication fork dynamics, PARylation balance, loss of PARP1, and drug efflux [33]. Many other resistance mechanisms are under study, and strategies to eliminating resistance or boosting the drug efficacy are also developing.

### 2.2. Check Point Inhibitors

Restoring anti-tumor immunity is a mainstay of cancer immunotherapy [34]. Programmed cell death 1 (PD-1) and programmed cell death ligand 1 (PD-L1) are well-known immune check points, and its ligand negatively regulates the cytotoxicity of anti-tumor effector cells. Several anti-PD-1 and anti-PD-L1 antibodies have been approved by the FDA for refractory melanoma and advanced NSCLC. Since TNBC is highly immunogenic with higher PD-L1 expression and immune-infiltration compared with luminal and HER2-enriched breast cancers, immunotherapy has been represented as a promising treatment strategy for TNBC [35,36]. Pembrolizumab is a human monoclonal IgG4-K antibody blocks the interaction between PD-1 and PD-L1/PD-L2. Pembrolizumab is undergoing clinical trials for TNBC. In a phase 1b KEYNOTE-012 trial, combination neoadjuvant chemotherapy and pembrolizumab for high-risk, early-stage TNBC showed foreseeable toxicity and promising anti-tumor activity with high pCR rates around 60% [37]. In both previously treated (cohort A) and untreated (cohort B) metastatic TNBC, pembrolizumab monotherapy showed a manageable safety profile and durable antitumor activity in a phase II KEYNOTE-086 study [38,39]. The results of the KEYNOTE-355 phase 3 trial also support adding pembrolizumab to standard chemotherapy for the first-line treatment of metastatic PD-L1-positive TNBC. Pembrolizumab–chemotherapy showed significant prolongation of PFS compared to that of the placebo–chemotherapy group [40]. Based on these data, the FDA approved pembrolizumab in combination with chemotherapy for patients with locally recurrent unresectable or metastatic TNBC whose tumors express PD-L1 in 2020. In July 2021, the FDA also granted pembrolizumab for high-risk, early-stage, TNBC in combination with chemotherapy as neoadjuvant treatment and continued as a single agent as adjuvant treatment after surgery. 

Another anti-PD-L1 monoclonal antibody, atezolizumab, has been approved by the FDA for combination with nab-paclitaxel in locally advanced or metastatic TNBC with higher expression of PD-L1. In a phase 1 study, atezolizumab’s single administration was well-tolerated in metastatic TNBC patients. In this study, the immune cell PD-L1 expression was independently associated with higher objective response rate and longer overall survival [41]. The combination of atezolizumab plus nab-paclitaxel for metastatic TNBC showed acceptable tolerance, with a 39.4% objective response rate in phase 1b trial regardless of previous treatment [42]. In a phase 3 Impassion130 trial, atezolizumab plus nab-paclitaxel as first-line treatment for locally advanced or metastatic TNBC prolonged progression-free survival compared to placebo plus nab-paclitaxel (7.2 months vs. 5.5 months, *p* = 0.002). This benefit was noticeable in the PD-L1-positive subgroup (7.5 months vs. 5.0 months, *p* < 0.001) [43]. Atezolizumab combined with neoadjuvant chemotherapy based on nab-paclitaxel and anthracycline also significantly improved the pCR rate of early TNBC patients in the Impassion031 phase 3 trial (*p* = 0.021) [44]. However, results of the IMpassion131 phase 3 trial, which evaluated atezolizumab combined with paclitaxel as first-line therapy for TNBC, failed to meet the primary objective of PFS superiority in the PD-L1-positive group (HR, 0.82; 95% CI, 0.60–1.12; *p* = 0.20). Neither OS advantage was observed in PD-L1-positive group (HR 1.11, 95% CI 0.76–1.64) [45]. According to this report, the indication for atezolizumab in combination with nab-paclitaxel as treatment for previously untreated, inoperable, locally advanced or metastatic PD-L1-positive TNBC has been withdrawn by Roche, the company responsible for the agent, following consultation with the FDA.

Other immune checkpoint inhibitors are also being investigated in clinical trials. CTLA-4 is expressed on activated T cells, which have affinity for CD80 and CD86 on antigen-presenting cells. Since CTLA-4 mediates immunosuppressive responses, inhibition of CTLA-4 is supposed to boost immune responses [46]. Ipilimumab is an anti-CTLA-4 antibody that is undergoing phase 2 clinical trial in combination with nivolumab (Clinicaltrials.gov: NCT03546686) [47].

### 2.3. Antibody-Drug Conjugates (ADC)

Antibody–drug conjugates are immuno-conjugate monoclonal antibodies to deliver high-potent cytotoxic small molecules to cancer cells. Sacituzumab govitecan is an anti-trophoblast cell-surface antigen (Trop-2) antibody conjugated with topoisomerase inhibitor. Trop-2 in breast cancer is known to be associated with poor prognosis [48]. Since Trop-2 is expressed in more than 90% of TNBC, the single-arm phase II clinical study proved the efficacy and safety of sacituzumab govitecan in heavily pretreated metastatic TNBC patients. The objective response rate was 30%, and treatment responses occurred early, with a median onset of 1.9 months [49]. Based on the data demonstrated in this trial, sacituzumab govitecan obtained the regular approval of the FDA for patients with unresectable, locally advanced, or metastatic TNBC who have received two or more prior systemic therapies, with at least one of them for metastatic disease. The recent phase III ASCENT trial reported significantly longer PFS and OS of sacituzumab govitecan compared to that of single-agent chemotherapy (median PFS 5.6 months with sacituzumab govitecan vs. 1.7 months with chemotherapy, *p* < 0.001; OS 12.1 months with sacituzumab govitecan vs. 6.7 months with chemotherapy, *p* < 0.001) [50].

## 3. Recently Developing Theranostics for Triple-Negative Breast Cancer

Although many target agents are under clinical trials, still, the number of therapeutic options for TNBC is the fewest among all breast cancer subtypes [51]. Systemic chemotherapy remains the mainstream of TNBC treatment. Recent advancements in nanotechnology are promising tools to overcome non-specific targeting and unwanted side-effect to healthy cells of chemo agents. Permeable size, adequate surface charge, and encapsulation capacity for the precise drug delivery, controlled release of drug, and minimum systemic toxicity with safe bio-elimination from the body are significant properties required for nanoparticles to be used as theranostics in cancer treatment [6]. Not only nanoparticles but ligands such as peptides or nucleotides are also being investigated for cancer nanomedicine. In this section, various nanoparticles are depicted and listed by their approaches.

### 3.1. Lipid-Based Nanoparticles

Lipid-based approaches have been spotlighted due to its hydrophobic characteristics with higher stability and biocompatibility [52]. Liposomes and solid lipid nanoparticles (SLN) are widely investigated for delivering therapeutic agents to the cancer site.

#### 3.1.1. Liposomes

Liposomes are 100–400 nm sized spherical vesicles with phospholipid bilayers encapsulating an aqueous core. Due to their feasibility to encapsulate either hydrophobic or hydrophilic drugs and their high efficacy, stability, non-immunogenic characteristics, and minimal systemic toxicity, Ref. [53] liposomes are versatile nanocarriers for drug delivery. Doxil^®^, comprised of doxorubicin encapsulated in STEALTH™ liposomes, received the U.S. FDA approval for Kaposi sarcoma or ovarian cancer treatment [54]. Su et al. designed poly ethylene glycol (PEG) engager, which simultaneously binds Doxil^®^ and epidermal growth factor receptor (EGFR) on TNBC cells [55]. Targeting EGFR has been widely investigated since Nogi et al. reported a higher incidence of EGFR overexpression (50%) among TNBC patients compared to that of luminal or HER-2 subtypes [56]. This PEG engager effectively promoted internalization of liposomal nanoparticles into target cells, which can enhance the antitumor activity of low-dose doxisome.

Andey et al. reported an 87% tumor growth inhibition effect of lipid nanocarriers of a lipid-conjugated estrogenic derivative in combination with cisplatin against TNBC as mice xenograft tumors [57].

The utilization of liposomes has been investigated not only in the chemotherapeutic field but also in the photodynamic therapy (PDT). Liposomes can deliver photosensitizers to the tumor, which can cause cytotoxicity after being triggered by a certain wavelength of light. Ding et al. combined PDT with a hypoxia-activated prodrug to produce a synergistic antitumor effect by using the PDT-induced hypoxic environment. They developed CD44-targeted liposomes encapsulating Photochlor as a photosensitizer and evofosfamide as the hypoxia-activated prodrug. CD44 effectively targeted TNBC cells, and the dual-loaded liposomes exhibited better antitumor activity compared to that of monotherapy groups [58]. 

Recently, nanosystems that co-encapsulate drugs in synergistic ratios have been widely investigated. This dual-drug-targeted approach using liposomes showed improved efficacy compared to that of free drugs. Franco et al. reported that a 1:10 co-encapsulation ratio of paclitaxel and doxorubicin in liposomes improved the cardiac toxicity profile in 4T1 murine breast cancer [59]. Dai et al. targeted integrin α3 using mTOR inhibitor rapamycin (RAPA) combined with doxorubicin (DOX)-loaded cyclic octapeptide liposomes. The synergetic effect between RAPA and DOX nanomedicine was confirmed in vitro and in vivo against a TNBC model [60].

#### 3.1.2. Solid Lipid Nanoparticles (SLN)

SLNs are made up of biodegradable physiological lipids with spherical particles ranging in size from 1 to 1000 nm. It is a promising colloidal carrier that can offer wide therapeutic and diagnostic application [61]. SLN has all the advantages of the liposomal system with avoidance of organic solvents use [62]. Several in vivo and in vitro studies reported increased intracellular drug accumulation in cancer cells and effective overcoming of drug efflux-mediated resistance of SLNs. SLN containing talazoparib produced by hot-homogenization technique showed significantly higher apoptotic rates in TNBC than free drug in vitro [63]. Abd-Ellatef et al. reported increased stability and intracellular retention of doxorubicin with curcumin (CURC)-loaded solid lipid nanoparticles in TNBC. CURC inhibits P-glycoprotein (Pgp), which is related to multidrug resistance. SLN enhances solubility and bioavailability of CURC, thus leading to effective bypass of Pgp-mediated doxorubicin resistance in TNBC cells [64]. SLN contains a gamma-secretase inhibitor (GSI) that has been also widely investigated. It is suggested that GSI inhibits epithelial-to-mesenchymal transition, angiogenesis, and tumor growth by blocking the notch signaling pathway [65,66]. The GSI-loaded SLNs conjugated with death receptor-5 or delta-like ligand 4 effectively blocked TNBC cell proliferation, differentiation, and metastasis with reduced off-target side effects [67,68]. 

### 3.2. Polymer-Based Nanoparticles

#### 3.2.1. Micelles

Polymeric micelles (PMs) are 10–100 nm sized colloidal particles with a hydrophobic core generated by the self-association of amphiphilic block copolymers [69]. PMs have received attention because of their marked stability, nanoscopic size, and biocompatibility arising from their solubility enhancement of hydrophobic drugs [70]. PMs also can increase chemosensitivity in cancer with multidrug resistance (MDR) through various mechanisms, such as inhibition of mitochondrial respiration and enhancing pro-apoptotic signaling in MDR cancer cells [71,72]. Zhao et al. reported the potency of polymeric micelle-based doxorubicin against free-doxorubicin-resistant TNBC stem cells. This PMs was formulated of doxorubicin with a Pluronic L61 and F127 mixture, which was named SP1049C. In previous study, SP1049C underwent phase II clinical trials and showed 43% of ORR in patients with advanced adenocarcinoma of the esophagus and gastro-esophageal junction [73]. Cancer stem cells (CSCs) are suggested to be associated with tumorigenesis, distant metastasis, and drug resistance [74]. Eradicating CSCs is challenging but is the critical issue in cancer treatment for preventing the cancer relapse. The authors reported that SP1049C effectively reduced the colony formation of CSCs compared to the free drug. Furthermore, doxorubicin/SP polymers showed greater cytotoxicity in CSCs than doxorubicin alone in vitro [71]. For better targeting of polymeric nanoparticles, Bressler et al. designed biomimetic peptide conjugated poly(lactic-co-glycolic acid)-block-polyethylene glycol (PLGA-PEG) nanoparticles. A collagen-IV-derived peptide, AXT050, which has high affinity to bind tumor-associated integrins, was conjugated to nanoparticles. In a murine model, biomimetic peptide-coated nanoparticles showed twofold greater cellular targeting ability compared to TNBC cells [75].

#### 3.2.2. Dendrimers

Dendrimers are branched-nanostructure polymers. The unique architecture of dendrimers, comprised of a central hydrophobic core and hydrophilic branched periphery, enables conjugation and attachments of molecules, especially genes such as siRNA [76]. They have high loading capacity of several functional groups simultaneously, which offers benefits for treating cancer. Moreover, rapid solubilization, biocompatibility, high permeability, and bioavailability of dendrimers are suitable features for their use as new drug-delivery agents [77]. Tomalia et al. first reported polyamidoamine dendrimers (PAMAM) in 1985 [78]. Throughout the generations, generation-4 PAMAM dendrimers (G4PAMAM) demonstrated high permeability across the epithelial cell monolayers with high efficiency for gene delivery in vivo [79]. Jain et al. investigated the delivery of polo-like kinase 1 (PLK1)-specific siRNA (siPLK1) conjugated to G4PAMAM. PLK1 is considered as a potential therapeutic target for the TNBC. The siPLK1 is reported to arrest the sub-G1 phase cells. G4PAMAM effectively protected the siPLK1 from degradation and delivered it to the TNBC cells. Intracellular uptake of siPLK1 in the TNBC cell line was enhanced in dendriplexes compared to that of naked siPLK1. Dendriplexes also caused increased cell arrest in the sub-G1 phase compared to siPLK1 solution [80]. Wang et al. reported that the G4PAMAM-VEGF-ASODN (antisense oligodeoxynucleotides) complex inhibits the tumor vascularization of a TNBC xenograft mouse model. G4PAMAM efficiently delivered ASODN into TNBC cells without significant cell toxicity [81]. The EGFR was validated as therapeutic target for TNBC compared to other types of breast cancer [82]. Liu et al. investigated the efficacy of the EGFR-targeting peptide and EGFR-binding peptide 1 (EBP-1). A dual-functional drug-delivery system based on PAMAM with EBP-1 and the cell-penetrating peptide encapsulating DOX significantly improved the anti-proliferation effect of the chemotherapeutic agent against TNBC cells in vitro [83]. Inoue et al. developed novel poly(β-L-malic acid) (PMLA)-based drug-delivery system that can directly target and inhibit EGFR. The authors used anti-tumor nucleosome-specific monoclonal antibody (mAb) 2C5 to target TNBC cells and Morpholino antisense oligonucleotide (AON) to inhibit EGFR synthesis. This nanobioconjugate showed stronger tumor growth inhibition in vivo [84].

#### 3.2.3. Quantum Dots (QDs)

Quantum dots are semiconductor nanoparticles with a symmetric emission band, long fluorescence lifetime, and strong photostability. QDs have been widely investigated in various biomedical areas, such as cellular imaging, cell trafficking, tumor targeting, and diagnostics [85,86]. Zheng et al. reported the advantages of quantum dot-based molecular pathology and quantitative detection of cancer cells over conventional IHC in breast cancer for the benefits of higher sensitivity and more accurate quantitative analyses. They performed in situ simultaneous imaging and quantitative detection of EGFR and collagen IV in TNBC using a QD-based multiplex molecular imaging algorithm. The EGFR/collagen IV ratio showed prognostic value for 5-DFS in TNBC [87]. 

EGFR-targeting QD-lipid nanocarriers (QLs) were also investigated by Park et al. for TNBC-targeted delivering of siRNA. The authors coupled the anti-EGFR aptamer to lipid nanocarriers containing anticancer siRNAs and quantum dots (aptamo-QLs). The vehicles were able to efficiently deliver siRNAs and fluorescent QDs to TNBC. The fluorescence signals by QDs enabled to analyze biodistribution of the lipid nanocarriers. Simultaneously, the delivered siRNA successfully inhibited tumor growth [88].

Black phosphorus quantum dots (BPQDs) are known to have effective photothermal effects with faster clearance. However, their instability and poor targeting ability have been considered as limitations. Nowadays, BPQDs coated with cancer cell membrane have been presented as a potential technological solution to solve these problems. Zhao et al. investigated cancer cell membrane-coated biomimetic BPQDs (BBPQDs) for treating TNBC. The capability of targeting TNBC cells was superior in BBPQDs than that of BPQDs, which was achieved through homologous targeting and tumor-homing effect. In addition, BBPQDs showed good direct photothermal cytotoxic effect along with indirect antitumor activity via dendritic cell maturation [89].

### 3.3. Carbon-Based Nanoparticles

#### Carbon Nanotubes (CNTs)

Carbon nanotubes are carbon allotropes with cylindrical nanostructure. They have received attention due to their large surface areas, rich surface chemical functionalities, high penetrating capability, and size stability [90]. CNTs have been used as vectors to deliver anticancer drugs as well as mediators for PTT and photodynamic therapy (PDT). There are three types of CNTs: single-wall carbon nanotubes (SWCNTs), multi-wall carbon nanotubes (MWCNTs), and double-wall carbon nanotubes (DWCNTs) [91]. Wailes et al. investigated MWCNTs for treating TNBCs. They mixed MWCNTs with collagen to replicate the structure of extracellular matrix, aiming to reprogram the cancer cell behavior by altering the cell’s mechanical environment. MWCNTs–collagen gels were able to restrict cell contraction, invasion, viability, MMP-9 expression, and migration of TNBC cells [92]. Zerda et al. demonstrated the potential of SWCNTs as photoacoustic molecular imaging agents in a TNBC-bearing mice model. Systemic administration of targeted SWCNTs showed excellent tumor accumulation and photoacoustic imaging ability in the mouse model [93]. Because of its intrinsic strong resonance Raman scattering and near infrared (NIR) photoluminescence with high optical absorbance, the dual application of SWCNTs as photoluminescent agents and photothermal tumor-eliminating agents has been widely investigated. Robinson et al. intravenously injected PEGylated SWCNTs to mice bearing 4T1 murine mammary tumors and achieved high spatial resolution NIR image of tumors in the 1.0–1.4 μm emission region. Further, complete tumor elimination was observed based on photothermal effect at NIR 808 nm without any toxic side effects [94].

## 4. Discussion

TNBC remains a challenging subtype of breast cancer with poor prognosis. The lack of expression of triple receptors makes it tough to treat. Systemic chemotherapy remains the standard treatment for TNBC. Anthracycline with cyclophosphamide (AC) followed by taxane (T) therapy is the standard adjuvant therapy for TNBC patients [95]. In relation to neoadjuvant chemotherapy, the INTENS trial reported that AC followed by T showed higher pCR rate and improved 5-year DFS compared to the TAC regimen [96]. The pCR rates vary among TNBC subgroups from 21.4% in the LAR subgroup to 65.6% in the BL1 subgroup [97]. For the recurrent or metastatic, currently available chemotherapies have limitations for treating TNBC due to the heterogeneity of disease. The drug toxicities and resistance are also associated with mortality. Nowadays, overexpression of several druggable target receptors of TNBC has been discovered. The rapid evolution of targeted therapy and immunotherapy is expected to improve the outcomes. A few monoclonal antibodies have been approved for clinical use of metastatic or refractory TNBC, such as PARP inhibitor, anti PD-L1 antibodies, and antibody drug conjugates. Several biomarkers have been under investigation for treating TNBC. However, further research is necessary to realize personalized medicine in TNBC. 

Besides targeting tumor cells itself, induction of anti-tumor immunity via targeting TME is one of the recently identified strategies for treating TNBC. Targeting TME has therapeutic advantage because of its stability and vulnerability compared to the genomic instability of cancer cells [98]. Various components of TME can affect the outcomes of immunotherapy. ECM can promote immunosuppression via impeding immune cell infiltration [99]. The presence of tumor-infiltrating lymphocytes (TIL), such as CD8+ and CD4+ T cells and natural killer (NK) cells, are known to correlate with favorable outcomes [100,101]. TNBC is known to be more immunogenic and immune-infiltrated than luminal or HER-2-enriched breast cancers [35]. According to this property, TNBC is more likely to benefit from immunotherapy. The accumulation of hyaluronan (HA) is associated with tumor growth and reduced overall outcomes of TNBC [102]. Remodeling the TME via degradation of HA can sensitize cancer cells to anti-PD-L1 immunotherapy [99]. Along with the development of immunotherapy, various attempts to oppose the immunosuppressive microenvironment of TNBC has been made. Further, large numbers of validation studies at clinical trials are necessary to confirm the efficacy of combination therapy.

Nanotechnology has gained attention for overcoming the limitations of conventional chemotherapy and systemic drug administration. Nanoparticles have been spotlighted in cancer research due to their target specific multifunctional characteristics. Lipid-based nanocarriers including liposomes and SLNs were able to deliver chemotherapeutic agents and photosensitizers with high biocompatibility. Polymeric-based delivery has advantages over lipid-based systems in the aspect of extended circulation time and higher accumulation inside tumor cells. Carbon-based nanoparticles such as CNTs are most versatile. In addition to having the advantages of both lipids and polymers, they possess conductive properties that can contribute to the detection and diagnosis of cancers. Successful designing of nanoparticles can be a promising approach for TNBC theranostics.

## Data Availability

Not applicable.
